# De-Differentiation Confers Multidrug Resistance Via Noncanonical PERK-Nrf2 Signaling

**DOI:** 10.1371/journal.pbio.1001945

**Published:** 2014-09-09

**Authors:** Catherine A. Del Vecchio, Yuxiong Feng, Ethan S. Sokol, Erik J. Tillman, Sandhya Sanduja, Ferenc Reinhardt, Piyush B. Gupta

**Affiliations:** 1 Whitehead Institute for Biomedical Research, Cambridge, Massachusetts, United States of America; 2 Department of Biology, Massachusetts Institute of Technology, Cambridge, Massachusetts, United States of America; 3 Koch Institute for Integrative Cancer Research, Cambridge, Massachusetts, United States of America; 4 Harvard Stem Cell Institute, Cambridge, Massachusetts, United States of America; 5 Broad Institute, Cambridge, Massachusetts, United States of America; St. Jude Children's Research Hospital, United States of America

## Abstract

Malignant carcinomas that recur following therapy are typically de-differentiated and multidrug resistant (MDR). De-differentiated cancer cells acquire MDR by up-regulating reactive oxygen species (ROS)–scavenging enzymes and drug efflux pumps, but how these genes are up-regulated in response to de-differentiation is not known. Here, we examine this question by using global transcriptional profiling to identify ROS-induced genes that are already up-regulated in de-differentiated cells, even in the absence of oxidative damage. Using this approach, we found that the Nrf2 transcription factor, which is the master regulator of cellular responses to oxidative stress, is preactivated in de-differentiated cells. In de-differentiated cells, Nrf2 is not activated by oxidation but rather through a noncanonical mechanism involving its phosphorylation by the ER membrane kinase PERK. In contrast, differentiated cells require oxidative damage to activate Nrf2. Constitutive PERK-Nrf2 signaling protects de-differentiated cells from chemotherapy by reducing ROS levels and increasing drug efflux. These findings are validated in therapy-resistant basal breast cancer cell lines and animal models, where inhibition of the PERK-Nrf2 signaling axis reversed the MDR of de-differentiated cancer cells. Additionally, analysis of patient tumor datasets showed that a PERK pathway signature correlates strongly with chemotherapy resistance, tumor grade, and overall survival. Collectively, these results indicate that de-differentiated cells up-regulate MDR genes via PERK-Nrf2 signaling and suggest that targeting this pathway could sensitize drug-resistant cells to chemotherapy.

## Abstract 2

Upregulation of PERK-Nrf2 signaling is a key mechanism by which de-differentiated cancer cells gain multi-drug resistance.

## Author Summary

The development of multidrug resistance is the primary obstacle to treating cancers. High-grade tumors that are less differentiated typically respond poorly to therapy and carry a much worse prognosis than well-differentiated low-grade tumors. Therapy-resistant cancer cells often overexpress antioxidants or efflux proteins that pump drugs out of the cell, but how the differentiation state of cancer cells influences these resistance mechanisms is not well understood. Here we used genome-scale approaches and found that the PERK kinase and its downstream target, Nrf2—a master transcriptional regulator of the cellular antioxidant response—are key mediators of therapy resistance in poorly differentiated breast cancer cells. We show that Nrf2 is activated when cancer cells de-differentiate and that this activation requires PERK. We further show that blocking PERK-Nrf2 signaling with a small-molecule inhibitor sensitizes drug-resistant cancer cells to chemotherapy. Our results identify a novel role for PERK-Nrf2 signaling in multidrug resistance and suggest that targeting this pathway could improve the responsiveness of otherwise resistant tumors to chemotherapy.

## Introduction

Multidrug resistance (MDR) is the primary obstacle to treating malignant tumors [1]. Cancer cells develop MDR by overexpressing antioxidant enzymes that neutralize the reactive oxygen species (ROS) required for chemotherapy toxicity or by up-regulating drug efflux pumps [2],[3]. In many cancers, these MDR mechanisms are up-regulated by mutation or amplification of genes encoding antioxidant enzymes or drug efflux pumps. Many other cancers, however, up-regulate these genes through nonmutational mechanisms that remain poorly understood.

One nonmutational mechanism by which cancer cells acquire MDR is de-differentiation. De-differentiation is a well-established marker of poor prognosis tumors and can occur when differentiated cells are induced into a more primitive stem-cell–like state [4]–[6]. One mechanism by which both cancerous and noncancerous cells can be de-differentiated is through induction of an epithelial-to-mesenchymal transition (EMT) [7]–[14]. De-differentiated cancer cells generated by EMT and cancer stem-like cells are both resistant to a wide range of chemotherapies [15]–[19]. Conversely, cells experimentally induced to differentiate are more sensitive to chemotherapies [20]–[23]. Although de-differentiation is known to up-regulate MDR mechanisms as described above, how this occurs is poorly understood.

In this article, we examine this question by employing a global transcriptional profiling approach to identify ROS-induced genes that are preactivated in de-differentiated cells. Many of these genes—which are activated in de-differentiated cells even in the absence of oxidative damage—are regulated by a single signaling pathway. We further show that this pathway is critical for de-differentiated cells to resist chemotherapies.

## Results

**Figure 1:**
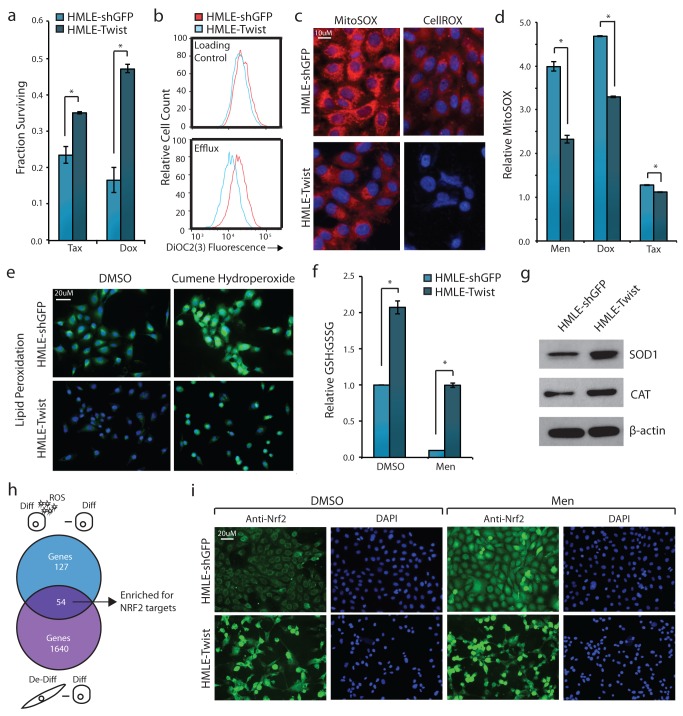
De-differentiated cells activate MDR and Nrf2 in the absence of oxidative or chemotherapy stress. (a) Fraction of HMLE-shGFP or HMLE-Twist cells surviving 3-d treatment with 2 nM Tax or 30 nM Dox, normalized to individual vehicle-treated controls. (b) Flow cytometry quantification of MDR1-mediated efflux ability. HMLE-shGFP or HMLE-Twist cells were loaded with cell-permeable DiOC2(3)-dye and efflux ability measured by loss of fluorescent signal after 1.5 h (efflux) compared to the loading control. (c) Fluorescent microscopy images of relative cellular ROS levels using the mitochondrial superoxide (MitoSOX) probe (red channel) or general oxidative stress (CellROX) probe (red channel) and cell nuclei labeled with DAPI (blue channel). (d) Flow cytometry quantification of MitoSOX fluorescence relative to individual vehicle-treated controls. Cells were treated with 40 µM menadione, 1 µM Dox, 1 µM Tax, or DMSO for 2 h prior to analysis. (e) Fluorescent microscopy images of relative lipid peroxidation levels (green channel) and cell nuclei labeled with DAPI (blue channel). Indicated cells were treated with 100 µM cumene hydroperoxide or DMSO for 2 h prior to analysis. (f) Relative amounts of reduced (GSH) to oxidized (GSSG) glutathione measured by luminescence-based assay. Indicated cells were treated with 40 µM menadione or DMSO for 2 h prior to analysis. Each sample is normalized to HMLE-shGFP DMSO control. (g) Western blot analysis of SOD1, CAT, and β-actin. (h) Overlap of genes >2-fold up-regulated in differentiated cells treated with 40 µM menadione (ROS) for 2 h compared to corresponding DMSO control (blue circle), and de-differentiated cells compared to differentiated cells in the absence of treatment (purple circle). (i) Immunofluorescence microscopy images of Nrf2 localization upon treatment with 40 µM menadione or DMSO for 2 h. Nrf2 protein was indirectly labeled with a secondary Alexa Fluor 488 antibody (green channel) and cell nuclei labeled with DAPI (blue channel). *p<0.05. Data are presented as mean ± SEM.

To study the effects of differentiation state on MDR, we used isogenic pairs of human breast epithelial cells (HMLE) that were either differentiated and expressed a control vector, or de-differentiated through induction of an EMT—achieved by expressing the Twist transcription factor [24],[25]. These de-differentiated HMLE-Twist cells were more resistant to the chemotherapy drugs Paclitaxel (Tax) and Doxorubicin (Dox) than differentiated HMLE-shGFP cells, consistent with prior reports (1.5× and 2.5×, respectively; Figure 1a) [26],[27]. To determine how Twist-induced de-differentiation caused MDR, we assessed whether known mechanisms were up-regulated in these cells. Twist overexpression significantly increased efflux pump activity (Figure 1b) and lowered ROS levels—both basal and induced by the oxidizer menadione or Dox (Figure 1c,d) [28]. Additionally, HMLE-Twist cells displayed significantly lower amounts of lipid peroxidation compared to HMLE-shGFP cells (Figure 1e). As a measure of overall reducing capacity of the cells, we also show that HMLE-Twist cells had a greater pool of reduced glutathione, which could be maintained even in the presence of menadione (Figure 1f). Finally, Twist overexpression led to a significant increase in expression of enzymes involved in ROS metabolism: superoxide dismutase 1 (SOD1) and catalase (CAT) (Figure 1g).

We suspected that these MDR mechanisms were up-regulated through a normal regulator of the cellular antioxidant response. To identify putative regulators, we transcriptionally profiled HMLE-shGFP and HMLE-Twist cells treated with vehicle or menadione (Table S1). In the absence of oxidative stress, 1,694 genes were differentially expressed between the two cell types, several of which were ROS and efflux-related genes (Tables S2 and S3). Treatment with menadione induced the expression of 181 and 170 genes in HMLE-shGFP and HMLE-Twist cells, respectively, with 44 genes being commonly induced in both cell types (Table S4; hypergeometric test, *p* value<1.0×10^−10^). Of the 181 genes induced by menadione in HMLE-shGFP cells, 54 were already up-regulated in HMLE-Twist cells in the absence of treatment (Table S5; hypergeometric test, *p* value<1.0×10^−10^, Figure 1h). Of these 54 genes, 38 were uniquely induced in HMLE-shGFP but not HMLE-Twist cells treated with menadione. This suggests that some oxidative stress response genes are “preactivated” in de-differentiated HMLE-Twist cells.

The most significantly preactivated gene in HMLE-Twist cells was heme oxygenase 1 (HMOX-1)—expressed at 8-fold higher levels in HMLE-Twist cells compared to HMLE-shGFP cells and induced 22-fold in differentiated cells treated with menadione. HMOX-1 is a well-characterized enzyme involved in the metabolism of heme, but is also a major target of master antioxidant regulator Nrf2 [29]–[31]. The Nrf2 transcription factor activates an arsenal of antioxidant genes and ABC transporters, and its up-regulation is associated with acquired MDR [32]–[35]. To test whether Nrf2 might be basally active in HMLE-Twist cells, but not HMLE-shGFP cells, we examined Nrf2 target gene expression. Of 1,013 Nrf2 direct-target genes, a significant number—142 genes—were up-regulated in HMLE-Twist cells compared to HMLE-shGFP cells in the absence of oxidative stress (Table S6; hypergeometric test, *p* value<1.0×10^−10^) [36]. Further, 7 of the 54 oxidative stress response genes “preactivated” in HMLE-Twist cells were Nrf2 direct-target genes, representing a significant enrichment over the number predicted by random chance (Table S5; hypergeometric test, *p* value = 4.9×10^−5^, Figure 1h). To confirm Nrf2 activation in HMLE-Twist cells, we assessed its subcellular localization by immunofluorescence. In HMLE-shGFP cells, Nrf2 was sequestered in the cytoplasm and translocated to the nucleus when cells were treated with menadione (Figure 1i). In HMLE-Twist cells, however, Nrf2 was constitutively in the nucleus, and treatment with menadione only modestly increased its nuclear accumulation (Figure 1i). These findings demonstrate that Nrf2 is constitutively active in de-differentiated HMLE-Twist cells—even in the absence of exogenous stress.

**Figure 2:**
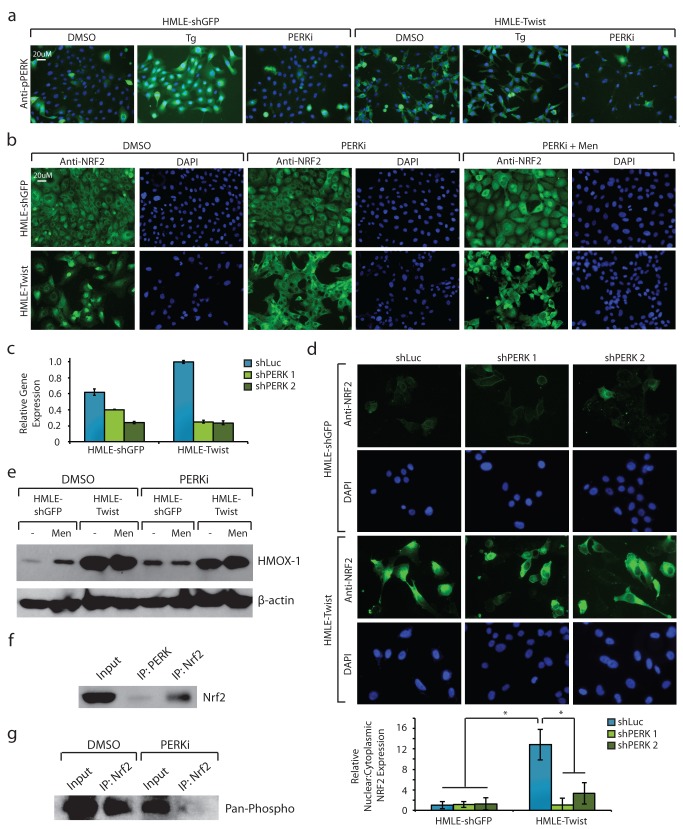
Nrf2 is constitutively activated by PERK in de-differentiated cells. (a) Immunofluorescence microscopy images of phospho-PERK (pPERK) upon treatment with 40 nM thapsigargin (Tg) for 2 h or 1 µM PERK inhibitor (PERKi) for 2 d. pPERK protein was indirectly labeled with a secondary Alexa Fluor 488 antibody (green channel) and cell nuclei labeled with DAPI (blue channel). (b) Immunofluorescence microscopy images of Nrf2 localization upon treatment with 1 µM PERKi or DMSO for 2 d, followed by 40 µM menadione or DMSO for 2 h. Nrf2 protein was indirectly labeled with a secondary Alexa Fluor 488 antibody (green channel) and cell nuclei labeled with DAPI (blue channel). (c) Quantitative RT-PCR analysis of PERK gene expression in cells stably expressing control (shLuc) or PERK-specific shRNA (shPERK 1 and shPERK 2). Expression is shown normalized to the HMLE-Twist shLuc sample. (d) Immunofluorescence microscopy images of Nrf2 localization in cell lines with stable knockdown of PERK compared to control knockdown cells. Nrf2 protein was indirectly labeled with a secondary Alexa Fluor 488 antibody (green channel) and cell nuclei labeled with DAPI (blue channel). Quantification of the number of cells with nuclear versus cytoplasmic Nrf2 localization is shown below. One hundred cells were analyzed per group, and the resulting ratio is normalized to the HMLE-shGFP shLuc group. (e) Western blot analysis of HMOX-1 and β-actin. HMLE-shGFP or HMLE-Twist cells were treated with 1 µM PERKi or DMSO for 2 d, followed by 40 µM menadione or DMSO for 2 h prior to cell lysis. (f) PERK or Nrf2-bound proteins were immunoprecipitated from HMLE-Twist cells, followed by immunoblotting for Nrf2. (g) Nrf2 immunoprecipitation followed by Western blot analysis of pan-phosphorylation. HMLE-Twist cells were treated with 1 µM PERKi or DMSO for 2 d prior to analysis.

We next examined why Nrf2 was constitutively active in HMLE-Twist cells, even though basal ROS levels are low. Although ROS activate Nrf2 by oxidation, it can also be activated in the absence of oxidative stress by several kinases [37]–[39]. In particular, Nrf2 is directly phosphorylated and activated by the ER-membrane kinase PERK, which is canonically activated under conditions of ER stress as part of the unfolded protein response (UPR) [40]–[42]. In this context, PERK relieves ER stress by slowing protein translation through phosphorylation of eiF2α. We have recently shown that PERK is also activated upon EMT-induced de-differentiation—even in the absence of overt ER stress [43]. Consistent with this, we found that PERK is constitutively phosphorylated in HMLE-Twist cells, but not in HMLE-shGFP cells, and inhibition of PERK with a small-molecule inhibitor blocked its phosphorylation (Figure 2a) [44]. To understand if PERK controls constitutive Nrf2 activation in HMLE-Twist cells, we assessed Nrf2 localization following PERK inhibition. We found that inhibition of PERK fully reversed the nuclear localization of Nrf2 in HMLE-Twist cells, but did not prevent oxidative stress-induced nuclear accumulation of Nrf2 in either HMLE-shGFP or HMLE-Twist cells (Figure 2b). As a complementary approach to PERK inhibition and to rule out off-target effects of the small-molecule PERK inhibitor, we also generated cell lines in which PERK expression was stably inhibited by two different shRNAs (Figure 2c). Inhibition of PERK by shRNA significantly decreased Nrf2 nuclear localization in HMLE-Twist cells, mirroring the results obtained with the small-molecule PERK inhibitor (Figure 2d). Collectively, these results demonstrate that Nrf2 nuclear localization is controlled by PERK in de-differentiated HMLE-Twist cells.

To confirm that Nrf2 nuclear localization correlated with its activation, we assessed Nrf2 target gene expression following PERK inhibition. We found that PERK inhibition significantly decreased HMOX-1 expression in HMLE-Twist cells, but did not prevent induction of HMOX-1 in response to oxidative stress (Figure 2e). Moreover, using microarray gene expression analyses, we found that PERK inhibition decreased the expression of 58 of the 142 Nrf2-target genes (41%) activated in HMLE-Twist cells (Table S6). Amongst these PERK-Nrf2-target genes were ABC transporters, enzymes involved in glutathione metabolism and ROS buffering, and several proteins with known roles in drug resistance. These findings confirm that the exit of Nrf2 from the nucleus correlates with down-regulation of its target genes.

PERK has previously been shown to bind to, directly phosphorylate, and activate Nrf2, though the exact phosphorylation sites have not yet been determined [42]. To show that PERK directly regulates Nrf2 in our system, we performed PERK immunoprecipitation followed by western blot with a Nrf2-specific antibody—which confirmed that PERK and Nrf2 directly interact in HMLE-Twist cells (Figure 2f). We also immunoprecipitated Nrf2 in either the presence or absence of the PERK inhibitor, which demonstrated that Nrf2 phosphorylation was markedly reduced by PERK inhibition (Figure 2g). These data, combined with our finding that inhibiting PERK decreases nuclear accumulation of Nrf2, suggest that PERK directly interacts with Nrf2 to mediate its nuclear translocation and activation.

**Figure 3:**
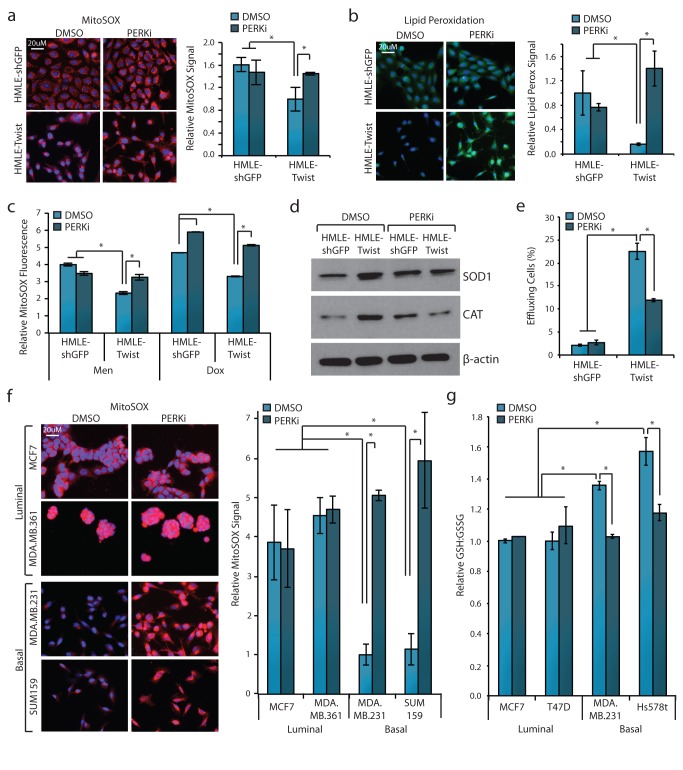
PERK activates MDR mechanisms in de-differentiated cells. (a) Fluorescent microscopy images of cellular ROS levels using the MitoSOX probe (red channel) and cell nuclei labeled with DAPI (blue channel). HMLE-shGFP or HMLE-Twist cells were treated with 1 µM PERKi or DMSO for 2 d prior to imaging. Quantification per cell is shown on the right. Each group is normalized to the HMLE-Twist DMSO group. (b) Fluorescent microscopy images of relative lipid peroxidation levels (green channel) and cell nuclei labeled with DAPI (blue channel). HMLE-shGFP or HMLE-Twist cells were treated with 1 µM PERKi or DMSO for 2 d prior to imaging. Quantification per cell is shown on the right. Each group is normalized to the HMLE-shGFP DMSO group. (c) Flow cytometry quantification of ROS buffering by measuring MitoSOX fluorescence relative to individual vehicle-treated controls. HMLE-shGFP or HMLE-Twist cells were treated with 1 µM PERKi or DMSO for 2 d, followed by 40 µM menadione, 1 µM Dox, or DMSO for 2 h prior to analysis. (d) Western blot analysis of SOD1, CAT, and β-actin. HMLE-shGFP or HMLE-Twist cells were treated with 1 µM PERKi or DMSO for 2 d prior to cell lysis. (e) Flow cytometry was utilized to quantitate efflux ability of HMLE-shGFP or HMLE-Twist cells treated with 1 µM PERKi or DMSO for 5 d. Results are shown as percentage of cells with the ability to efflux >50% of loaded DiOC2(3)-dye. (f) Fluorescent microscopy images of cellular ROS levels in luminal and basal breast cancer cell lines using the MitoSOX probe as in (a). Each group is normalized to the MDA.MB.231 DMSO group. (g) Relative amounts of reduced (GSH) to oxidized (GSSG) glutathione. Luminal and basal breast cancer cells were treated with 1 µM PERKi or DMSO for 2 d prior to analysis. Each sample is normalized to the MCF7 DMSO group. *p<0.05. Data are presented as mean ± SEM.

We next tested whether inhibition of PERK would eliminate MDR phenotypes associated with HMLE-Twist cells. PERK inhibition caused a 45% increase in mitochondrial ROS levels in HMLE-Twist cells, but did not affect HMLE-shGFP cells (Figure 3a). PERK inhibition also significantly increased lipid peroxidation in HMLE-Twist cells, but not in HMLE-shGFP cells (Figure 3b). PERK inhibition compromised ROS buffering—cells pretreated with the PERK inhibitor produced 25%–55% more ROS than vehicle-treated cells (Figure 3c). Additionally, PERK inhibition led to a significant decrease in the expression of ROS metabolizing enzymes SOD1 and CAT (Figure 3d). Lastly, inhibition of PERK signaling reduced the percentage of high-effluxing HMLE-Twist cells by 50% and did not affect efflux in HMLE-shGFP cells (Figure 3e). Together these results demonstrate that a simple change in differentiation state confers MDR phenotypes, and these are mediated by constitutive PERK signaling.

To understand how this applies in the context of cancer, we expanded our analyses to include several luminal and basal-like breast cancer cell lines, which represent epithelial-like/differentiated and mesenchymal-like/de-differentiated cells, respectively [45]. Previous work has shown that PERK is preferentially activated in basal compared to luminal cell lines [43]. Consistent with our results in the HMLE system, basal breast cancer cells had lower overall ROS than luminal cells, and addition of the PERK inhibitor caused a dramatic increase in ROS levels in basal cells but not luminal cells (Figure 3f). Likewise, inhibition of PERK caused a 25% reduction in the ratio of reduced to oxidized glutathione in only the basal cell lines, indicative of decreased ROS buffering (Figure 3g). This indicates that PERK contributes to the enhanced oxidative stress buffering ability of both noncancerous and cancerous de-differentiated cells.

**Figure 4:**
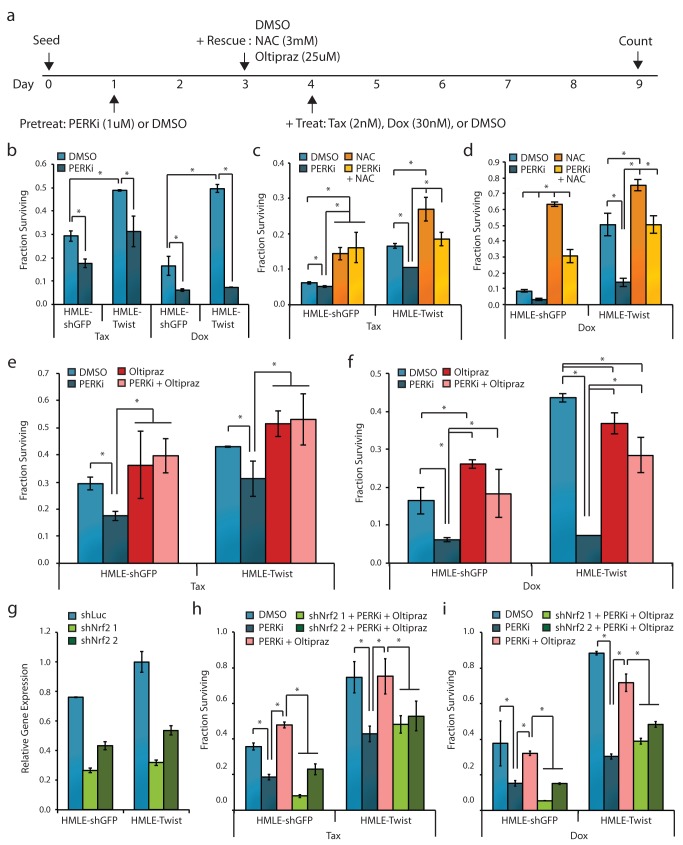
Inhibition of PERK-Nrf2 signaling sensitizes de-differentiated cells to chemotherapy. (a) Schematic of treatment timing and dosage for cell survival experiments described in (b–f). (b) Fraction of cells surviving 2 nM Tax or 30 nM Dox following pretreatment with 1 µM PERKi or DMSO, normalized to individual vehicle-treated controls. (c and d) Fraction of cells surviving 30 nM Dox (c) or 2 nM Tax (d) following pretreatment with 1 µM PERKi or DMSO and rescue with 3 mM NAC, normalized to individual vehicle-treated controls. (e and f) Fraction of cells surviving 30 nM Dox (e) or 2 nM Tax (f) following pretreatment with 1 µM PERKi or DMSO and rescue with 25 µM Oltipraz, normalized to individual vehicle-treated controls. (g) Quantitative RT-PCR analysis of Nrf2 gene expression in cells stably expressing control (shLuc) of Nrf2-specific shRNA (shNrf2 1 and shNrf2 2). Expression is shown normalized to the HMLE-Twist shLuc sample. (h and i) Fraction of control or Nrf2 knockdown cells surviving 30 nM Dox (h) or 2 nM Tax (i) following pretreatment with 1 µM PERKi or DMSO and rescue with 25 µM Oltipraz, normalized to individual vehicle-treated controls. *p<0.05. Data are presented as mean ± SEM.

In order to affect chemotherapy resistance, we rationalized that PERK inhibition would need to occur prior to chemotherapy exposure to allow time for reversal of MDR phenotypes (Figure 4a). Pretreatment with the PERK inhibitor greatly sensitized both HMLE-Twist and HMLE-shGFP cells to subsequent treatment with Tax and Dox—the number of surviving cells was reduced significantly in both cell types (Figure 4b). Treatment with a ROS-scavenging agent n-acetyl cysteine (NAC) was able to rescue this decreased survival, indicating that PERK pathway activation contributes to chemotherapy resistance in significant part via ROS buffering (Figure 4c,d) [46]. We also utilized a small molecule—oltipraz—capable of inducing Nrf2 activation (Figure 4e,f) [47]. Activation of Nrf2 significantly rescued PERK-dependent decreases in cell survival. To rule out the possibility that off-target effects of oltipraz were responsible for this effect, we performed the same rescue experiment in cells with stable Nrf2 knockdown achieved by two independent shRNAs. When Nrf2 was inhibited, oltipraz was no longer able to rescue the effects of PERK inhibition, confirming that these effects were mediated by Nrf2 (Figure 4g–i). These results indicate that PERK signaling through Nrf2 is responsible for the acquisition of MDR.

**Figure 5:**
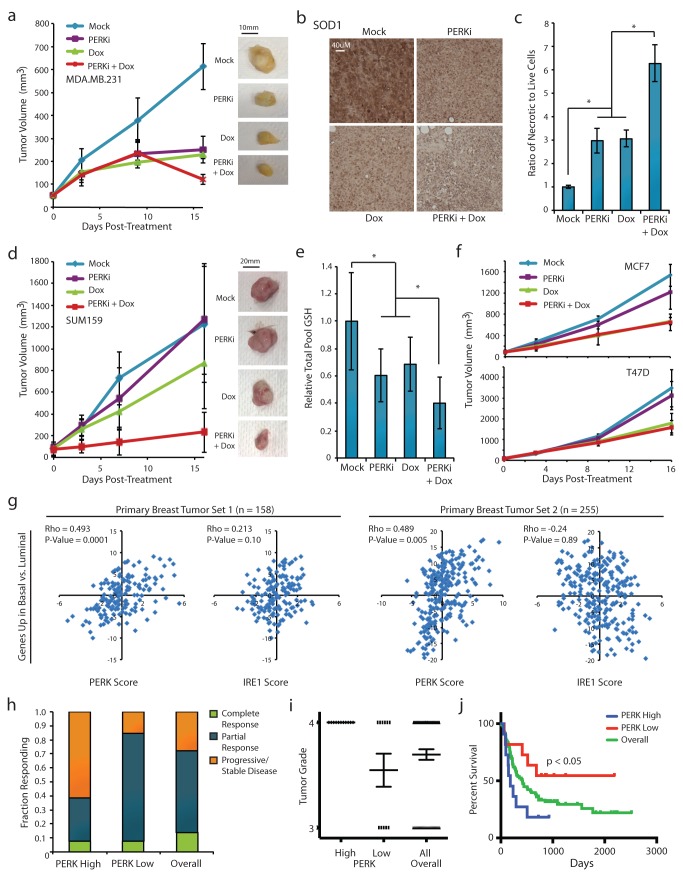
PERK promotes chemotherapy resistance in vivo and correlates with tumor de-differentiation in patient samples. (a) MDA.MB.231 cells were injected bilaterally into the mammary fat pads of female NOD/SCID mice. After reaching 60–80 mm^3^, tumors were treated with PERKi (7.5 mg/kg/tumor) or DMSO by intratumoral injection, and Dox (2.5 mg/kg) or PBS by intraperitoneal (IP) injection according to the schedule described in Materials and Methods. Tumor volume over time with images of representative tumors is shown. (b) Formalin-fixed paraffin-embedded tumor sections were stained for SOD1, and nuclei were counterstained with hematoxylin. Representative images are shown for each treatment group. (c) Ratio of necrotic to live cells per tumor, presented as the average across tumors for each group. (d) SUM159 cells were injected as above and tumor growth was monitored. The treatment schedule was modified to reduce the frequency of treatments to days 1, 2, 8, and 9. Tumor volume over time with images of representative tumors is shown. (e) Relative amount of reduced glutathione (GSH) per tumor for each treatment group, shown normalized to the mock treatment group. (f) MCF7 or T47D cells were injected as above and tumor growth was monitored. The treatment schedule was modified to reduce the frequency of treatments to days 1, 2, 8, and 9. Tumor volume over time is shown. (g) Correlation analyses of PERK pathway genes or IRE1 pathway genes and genes up-regulated in basal compared to luminal breast tumors. Analyses were performed for two primary breast tumor datasets (GSE3143, *n* = 158; GSE41998, *n* = 255). Spearman's rho (Rho) was used to measure correlation. (h) Fraction of PERK-high and PERK-low primary breast tumors showing complete response, partial response, or stable/progressive disease upon treatment with standard AC regimen plus paclitaxel. (i) Scatter dot plot depicting the distribution of PERK-high and PERK-low primary human glioma tumors classified as grade 3 or grade 4. Line represents mean ± SEM. (j) Kaplan-Meier plots of distant metastasis-free survival of glioma patients. Patient groups were separated based on PERK-high or PERK-low signature expression and shown compared to overall survival. The Mantel-Cox test was utilized to determine the significance of the survival difference between PERK-high and PERK-low patient groups. *p<0.05. Data are presented as mean ± SEM.

These results prompted us to test the effect of PERK inhibition in vivo, utilizing xenografted tumors derived from therapy-resistant basal breast cancer cells. We utilized a treatment plan involving cycles of pretreatment with the PERK inhibitor, followed immediately by treatment with Dox. The combined treatment resulted in significantly smaller tumors compared to single or mock treatments (Figure 5a). To test if PERK inhibition affected ROS buffering in vivo, we harvested tumors from each of the four treatment groups and measured the expression of the ROS-metabolizing enzyme SOD1. We found that the Dox, PERK inhibitor, and combined treatment groups all had significantly reduced expression of SOD1 compared to control tumors, with the dual-treated tumors having the lowest expression (Figure 5b). Additionally, the combined treatment group had the most necrotic cells compared to the other treatment groups (Figure 5c).

We next adjusted the dosage schedule to highlight the synergistic interactions between PERK inhibition and Dox treatment and found that reducing the total dosage and frequency of treatments further emphasized the sensitization effect—dual-treated tumors were 4 times smaller than Dox-treated tumors and >5 times smaller than PERK inhibitor or mock-treated groups (Figure 5d). Although prior research has shown that PERK is critical for tumor growth and angiogenesis [48]–[50], we found that low-dose inhibition only minimally impacted tumor growth in the absence of chemotherapy. To assess the in vivo effects on ROS buffering, we measured the levels of reduced glutathione (GSH) in tumors harvested from each treatment group. Dox, PERK inhibitor, and combined treatment groups all had decreased levels of GSH compared to the control group, with the dual-treated tumors having the lowest amount (Figure 5e).

As an important control to demonstrate that the observed in vivo results were not due to off-target effects of the PERK inhibitor, we utilized xenografted tumors derived from luminal breast cancer cells. Although treatment with Dox led to a reduction in tumor size, inhibition of PERK did not provide any additive benefit in the luminal tumors (Figure 5f). This confirms that the effects observed in the basal breast cancer xenografts are not due to off-target effects of the PERK inhibitor, as luminal cells—unlike basal cells—do not constitutively activate PERK and do not significantly respond to PERK inhibition. Together our results suggest that combining Dox treatment with PERK inhibition compromises the ROS-buffering capacity of basal-like breast cancer cells and sensitizes them to chemotherapy-induced cell death.

To assess the clinical relevance of our findings, we analyzed primary human breast tumor datasets. Utilizing two independent datasets (comprised of 413 patient tumors), we first tested for correlations between the expression of PERK pathway genes and genes associated with the basal subtype of breast cancer. We found that a PERK gene expression signature correlated positively with a basal breast cancer gene signature, suggesting that the PERK signaling pathway is active in basal breast tumors (Figure 5g) [51]. As a negative control, an IRE1 gene expression signature did not show a significant correlation (Figure 5g). Additionally, we found that PERK pathway activity could stratify patient response to therapy—85% of PERK-low tumors displayed complete or partial response to therapy, compared to only 38% of PERK-high tumors (Figure 5h). Finally, PERK pathway expression also correlated to differentiation state and overall survival in invasive high-grade glioma—tumors stratified into a PERK-high group were almost exclusively poorly differentiated grade 4 GBM and had significantly worse overall survival than the PERK-low group (Figure 5i,j). These results highlight the relevance of our work in primary tumors, and suggest that targeting PERK signaling may be beneficial in highly aggressive and malignant tumor types.

## Discussion

These findings identify PERK-Nrf2 signaling as one mechanism by which de-differentiated cells gain MDR. Because they constitutively activate Nrf2, these de-differentiated cells constitutively express antioxidant enzymes and drug efflux pumps. Remarkably, in this setting, Nrf2 is not activated by oxidation, but rather through a previously reported mechanism involving its phosphorylation by PERK [42]. This finding is of particular interest given Nrf2's known role in promoting chemotherapy survival [34] and its constitutive activation by mutation in a subset of tumors [52]–[54]. Our findings indicate that a change in cellular state, in the absence of mutation or oxidative stress, can also lead to constitutive Nrf2 activation. This enables de-differentiated cells to survive chemotherapy by preventing cellular damage before it occurs. In contrast, differentiated cells activate Nrf2 only after proteins and DNA have been oxidized. Although this defensive response may succeed in neutralizing toxins, the damage to cellular components would have already occurred.

Our findings also highlight the importance of stress signaling in cancer. Cancer cells activate stress response pathways to protect themselves from harsh environments encountered during tumor growth and metastasis—for example, hypoxia and nutrient deprivation—and also during the course of chemotherapy. We show that de-differentiated tumor cells preactivate PERK-Nrf2 signaling in the absence of stress and that inhibition of PERK sensitizes these cells to chemotherapy. These observations complement prior studies establishing a role for the UPR and its downstream targets in chemosensitization [55]–[67]. Collectively, our findings provide mechanistic insights into how cellular de-differentiation promotes MDR and suggest that inhibiting PERK-Nrf2 signaling may reverse the MDR of cancer cells that are otherwise drug resistant.

## Materials and Methods

### Ethics Statement

This study was performed in strict accordance with the recommendations in the Guide for the Care and Use of Laboratory Animals of the National Institutes of Health. The protocol was approved by the Animal Care and Use Committee of the Massachusetts Institute of Technology (Protocol No. 0611-071-14). All surgery was performed under isoflurane anesthesia, and every effort was made to minimize suffering.

### Cell Lines and Reagents

HMLE-shGFP and HMLE-Twist cell lines were a kind gift of Dr. Robert Weinberg and cultured as described previously [8]. Basal breast cancer (MDA.MB.231, Hs578t) and luminal breast cancer (MCF7, T47D, MDA.MB.361) cell lines were purchased from ATCC and cultured in DMEM+10% FBS. The SUM159 basal breast cancer cell line was purchased from Asterand and cultured in Ham's F-12+5% FBS, Insulin and Hydrocortisone. Chemical oxidizer menadione, Nrf2 activator oltipraz, and ER-stress inducer thapsigargin were purchased from Sigma-Aldrich. Cumene hydroperoxide (CH) was purchased from Life Technologies. The PERK inhibitor (PERKi) was described previously and purchased from EMD Millipore [44]. Lentiviral short hairpin RNA (shRNA) constructs were generated as described previously [68]. Lentiviral integration was selected with 1 µg/ml puromycin or 10 µg/ml blasticidin for 7 d, and knockdown efficiency was measured by quantitative RT-PCR.

### ROS Assays

ROS production was measured by fluorescent imaging or flow cytometry analysis of MitoSOX or CellROX probes (Life Technologies) according to manufacturer instructions. Lipid peroxidation was assessed using the Click-iT Lipid Peroxidation Imaging Kit (Life Technologies) according to the manufacturer instructions. Total, reduced, and oxidized glutathione were determined using the GSH/GSSG-Glo™ Assay (Promega) according to manufacturer instructions.

### Efflux Assays

MDR1 efflux ability was measured by flow cytometry quantification of DiOC2(3)-dye efflux (EMD Millipore). Efflux assays were conducted according to manufacturer instructions. Briefly, cells were loaded with DiOC2(3)-dye for 10 min and either kept on ice or placed at 37°C for 1.5 h to allow efflux of the dye. Control and efflux samples were then immediately analyzed by flow cytometry.

### Western Blot

Cells were lysed with cold RIPA buffer plus complete protease inhibitor cocktail (Roche Applied Science). The signal was detected using the SuperSignal ECL system (Thermo Scientific). The following antibodies were used for immunoblotting: SOD1 and Nrf2 (Santa Cruz Biotechnology) and CAT, HMOX-1, pan-phospho, and β-actin (Cell Signaling Technologies).

### Immunoprecipitation

HMLE-Twist cells grown in the presence or absence of 1 µM PERK inhibitor for 48 h were lysed in nondenaturing lysis buffer (20 mM Tris pH 7.5, 150 mM NaCl, 2 mM EDTA, 1% NP-40, supplemented with cocktails for phosphatase and protease inhibition). Equal protein amounts were used for immunoprecipitation using PERK or Nrf2 antibody as per the vendors' instructions. Samples were analyzed by immunoblotting using antibodies to Nrf2 and phospho-Ser/Thr–containing proteins.

### Immunofluorescence

Anti-Nrf2 (C-2) and anti–phospho-PERK (pPERK) antibodies was purchased from Santa Cruz Biotechnology. Cells were fixed on glass chamber slides in 4% PFA for 5 min, blocked with 5% BSA in PBS, and incubated with primary antibody at a 1∶50 dilution for 2 h. Slides were then washed with PBS and incubated with an Alexa Fluor 488 anti-rabbit secondary antibody. The nuclei were then stained with DAPI prior to analysis.

### Immunohistochemistry

Immediately after harvest, tumors were fixed in 4% PFA for 24 h and paraffin-embedded. For staining, slides were deparaffinized in xylene and then rehydrated with ethanol and double distilled water. Hydrogen peroxide was used to block nonspecific sites, and Diva Decloaker (BioCare Medical) solution and microwaving were used for antigen retrieval. Sections were incubated for 2 h at room temperature with a SOD1 antibody (Santa Cruz Biotechnology). Expression was detected using HRP anti-rabbit secondary antibody (BioCare Medical) and betazoid DAB (BioCare Medical). The slides were counterstained with hematoxylin.

### Microarray Analysis

HMLE-shGFP and HMLE-Twist cells were treated with 1 µM of PERKi or DMSO for 48 h and then treated with 40 µM menadione or DMSO for 2 h immediately before RNA extraction. Total RNA were extracted using Qiagen RNeasy kit, and integrity and quality verified prior to analysis. Gene expression analyses were conducted using Affymetrix GeneChip Human Genome U133 Plus 2.0 Arrays according to standard Affymetrix protocols, with normalization as described previously [69]. Alteration of gene expression by PERKi and/or menadione was calculated by comparing the expression of each gene across treatment groups for each cell type. The gene-expression data have been deposited in the NCBI Gene Expression Omnibus public database (GEO; GSE59780).

### Cell Survival Experiments

Cells were seeded and treated according to the schedule described in Figure 4a. Briefly, cells were seeded on day 0, pretreated with PERKi (1 µM) or DMSO for 48 h on days 1–3, and rescued with NAC (3 mM), oltipraz (25 µM), or DMSO for 24 h on day 3. Cells were then treated with Dox (30 nM), Tax (2 nM), or DMSO on day 4. Cells were followed for an additional 5 d, with complete media and drug replacement on day 7. Cell survival was assessed on day 9 by manual cell count and normalized as described for each experiment.

### Animal Experiments

Female NOD/SCID mice were purchased from Jackson Labs. The Animal Care and Use Committee of the Massachusetts Institute of Technology approved all animal procedures. For tumor regression studies, 1×10^6^ cells were injected bilaterally into the mammary fat pad of 6–8-wk-old female NOD/SCID mice. After reaching 60–80 mm^3^, tumors were treated with PERKi (7.5 mg/kg/tumor) or DMSO by intratumoral injection on days 1, 2, 4, 5, 8, 9, 11, and 12, and Dox (2.5 mg/kg) or PBS by intraperitoneal (IP) injection on days 2, 5, 9, and 12 unless otherwise specified. Tumor volume over time and average tumor weight at sacrifice were measured and presented as the average ± standard error of mean for 10 tumors per treatment group.

### Correlation Analysis of Gene Expression for Human Cancer Samples

For correlation analyses, the PERK gene expression signature was defined as the top 500 genes down-regulated in de-differentiated cells treated with 1 µM PERKi for 48 h. The PERK signature scores were calculated for each patient sample from human breast cancer (GSE3143, GSE41998) and glioma (GSE4412) datasets by summing the log-transformed normalized expression values for each probe in the signature set. High- and low-PERK signature groups were defined as the top or bottom 15% of samples within each group. Spearman's rho was used to measure correlation, and a *p* value was determined by Monte Carlo sampling as described previously [43].

### Statistical Analysis

All data are presented as mean ± standard error of mean unless otherwise specified. Student *t* test (two-tailed) was used to calculate *p* values, and *p*<0.05 was considered significant.

## Supporting Information

Table S1 **Normalized gene expression data.**
(XLSX)

Table S2 **Genes differentially expressed between HMLE-Twist and HMLE-shGFP cells.**
(XLSX)

Table S3 **ROS and efflux-related genes up-regulated in HMLE-Twist compared to HMLE-shGFP cells.**
(XLSX)

Table S4 **Genes up-regulated upon treatment with menadione.**
(XLSX)

Table S5 **ROS-responsive genes pre-activated in HMLE-Twist cells.**
(XLSX)

Table S6 **Differentially expressed Nrf2 target genes.**
(XLSX)

## Abbreviations

CAT: catalase
Dox: doxorubicin
EMT: epithelial-to-mesenchymal transition
GSH: reduced glutathione
GSSG: oxidized glutathione
HMLEs: human mammary epithelial cells
HMOX1: heme oxygenase 1
MDR: multidrug resistance
NAC: n-acetyl cysteine
ROS: reactive oxygen species
SOD1: superoxide dismutase 1
Tax: paclitaxel
UPR: unfolded protein response
